# Essential Oil Composition and Larvicidal Evaluation of *Platycladus orientalis* against Two Mosquito Vectors, *Anopheles stephensi* and *Culex pipiens*

**Published:** 2018-06-13

**Authors:** Alireza Sanei-Dehkordi, Sahereh Gholami, Mohammad Reza Abai, Mohammad Mehdi Sedaghat

**Affiliations:** 1 Department of Medical Entomology and Vector Control, Faculty of Health, Hormozgan University of Medical Sciences, Bandar Abbas, Iran; 2 Infectious and Tropical Diseases Research Center, Hormozgan Health Institute, Hormozgan University of Medical Sciences, Bandar Abbas, Iran; 3 Department of Medical Entomology and Vector Control, School of Public Health, Tehran University of Medical Sciences, Tehran, Iran

**Keywords:** *Anopheles stephensi*, *Culex pipiens*, *Platycladus orientalis*, Essential oil, Larvicide

## Abstract

**Background:**

Natural plant products as larvicides could be considered as desirable alternatives to synthetic chemical insecticides for vector management. This study was undertaken to assess the mosquito larvicide activity of the essential oil from fresh leaves of *Platycladus orientalis* against two medically important species of mosquito vectors.

**Methods:**

Essential oil was extracted by hydrodistillation and analyzed with gas chromatography and mass spectrometry (GC-MS). Fresh leaves of *P. orientalis* tree (500g) were collected in June 2014 from Tehran, Iran and was authenticated at the Department of Medical Entomology and Vector Control, School of Public Health, Tehran University of Medical Sciences, Tehran, Iran.

In addition, the larvicidal potential of oil was evaluated against late-3^rd^ or young-4^th^ instar larvae of *Anopheles stephensi* and *Culex pipiens* under laboratory condition. The mortality counts were made after 24h and LC_50_ and LC_90_ values were calculated.

**Results:**

Forty-six components in leaves of *P. orientalis* were identified. The major components were α-Pinene (20.17%), 3-Carene (14%) and Cedrol (9.51%). The LC_50_ values against *An. stephensi* and *Cx. pipiens* larvae were 11.67ppm and 18.60ppm after 24h, respectively.

**Conclusion:**

*Platycladus orientalis* oil could be considered as a natural larvicide for mosquito larval control.

## Introduction

Mosquitoes are the most important group of arthropods with medical importance, which can transmit many pathogens and parasites cause important diseases such as malaria, dengue, yellow fever and filariasis throughout the world, except the Antarctic (1). Human malaria caused by protozoans (*Plasmodium* spp.) continues to be the most important vector-borne disease. It affects more than 91 tropical countries, placing 3.2 billion people at risk as of 2015. Overall, 212 million cases of malaria and 429000 deaths were reported worldwide (2).

This disease is transmitted only by the anopheline mosquitoes. There are more than 400 described species of *Anopheles* worldwide, from which about 40 species are important vectors of human malaria (3). In Iran, malaria is transmitted by seven vector species, among these species, *Anopheles stephensi* is considered as a primary vector of malaria in the southern parts of Iran (4–6).

*Culex* species are important vectors of human pathogens worldwide including the etiologic agents of different forms of encephalitis, Rift valley fever, and lymphatic filariasis. *Culex pipiens* with broad global distribution is considered as one of the most medically important vectors (1).

There are several strategies applied for mosquito control, the usage of synthetic larvicides still remains as an effective method. The indiscriminate use of synthetic insecticides such as organophosphorus compounds have several adverse effects on environment, toxic effects on human beings and non-target organism (specifically aquatic insects) and also may lead to the development of resistance in mosquito populations (7–9).

The current trend has highlighted the search for new compounds for mosquito larval control. From this viewpoint, botanical insecticides are promising since they are effective, environmentally friendly, degradability, non-toxic effects on non-target organisms and often inexpensive. Aromatic plants and their essential oils have been suggested as natural insecticides for pest control because they have few harmful effects on ecosystem structure and function (10). The insecticidal properties of plant volatile oils and extracts from a wide variety of plants have been assessed against the larvae of different species of mosquito (11–14).

*Platycladus orientalis* (Linnaeus) Franco [synonym: *Thuja orientalis* Lineous, *P. stricta* Spach, *Biota orientalis* locally named as Sarv-e Khomrei or Noosh, is a monoecious and evergreen tree belonging to the Labiatae family which grows wild in Korea, Japan, China, and Iran. This plant is cultivated as a common ornamental plant in different parts of Iran and other countries (15).

*Platycladus orientalis* is used in Iranian traditional medicine as an astringent, stomach tonic, diuretic, tonic and antipyretic effect (16). The essential oil of *P. orientalis* has been evaluated for antimicrobial activity (17), antifungal activity (18, 19), cytotoxicologic activity (20), molluscicidal activity (21) and insecticide activity (22, 23).

The main purpose of this study was to analyze the essential oils by GC-MS in order to identify the constituents and larvicidal activity of the leaf essential oil of *P. orientalis*.

## Materials and Methods

### Plant materials

Fresh leaves of *P. orientalis* tree (500g) were collected in June 2014 from Tehran, Iran (51° 23’E, 35° 42’N, elevation: 1242m). The plant was identified and authenticated at Department of Medical Entomology and Vector Control, School of Public Health, Tehran University of Medical Sciences, Tehran, Iran.

### Essential oil isolation

The fresh leaves of *P. orientalis* were cut into small pieces and subjected to hydrodistillation using Clevenger-type apparatus (Model: British Pharmacopoeia, manufactured by Pyrexfan Company, Iran and mantle model EM manufactured by Bibby Scientific Company, United Kingdom). The essential oil was dried over anhydrous sodium sulfate and the purified oil transferred into amber-colored vials at +4 °C until further use.

### Gas chromatography/mass spectrometry

The oil analysis was carried out using GC-MS. The GC apparatus was Agilent technology (HP) 6890 system, capillary column of HP-1MS (Fused silica) (30m× 0.25mm, film thickness 0.25μm). The oven temperature program was initiated at 40 °C, held for 1 min then raised up to 230 °C, rising to 250 °C at 5 °C/min. The carrier gas used was helium at a flow rate of 1ml/min. The detector and injector temperatures were 260 °C and 230°C, respectively. GC/MS analysis was conducted on an HP 6890 GC system coupled with a 5973 network mass selective detector with a capillary column the same as above, carrier gas helium with flow rate 1 ml/min. A sample of 1μL was injected in the split mode with split ratio 1:50. For GC–MS detection, an electron ionization system, with ionization energy of 70eV, was used, injector and oven temperature programmed was identical to GC. The Retention indices (RI) were calculated for all volatile constituents using a homologous series of n-alkanes (C_8_–C_24_) on the HP-1MS column. Quantification was performed using percentage peak area calculations and the identification of individual compartments was done using the Wiley 7n.1 GC-MS library, NIST (National Institute of Standards and Technology) and those published in the literature (24–26).

### Mosquito rearing

The larvae of *An. stephensi* and *Cx. pipiens* were obtained from the anopheline insectary of Department of Medical Entomology, Tehran University Medical Sciences. The continuous breeding colony of mosquito was maintained at 27 °C with 12:12 L/D photoperiod in 65±5% RH. Larvae of *An. stephensi* were available for the mosquito larvicidal experiments.

### Bioassays and larval mortality

Bioassay tests were performed according to the standard method recommended by WHO (27). In brief, the essential oil first dissolved in absolute ethanol. A 400ml glass beaker was used for each experiment or control. The late-3^rd^ or young-^4th^ instar larvae of *An. stephensi* and *Cx. pipiens* were exposed to 5, 10, 20, 40 and 80ppm of essential oil in water, in the control beakers only solvent (absolute ethanol) was dissolved into the water. Mortality was counted after 24h. When necessary, mortality was adjusted for control deaths using Abbott’s formula (28). The lethal concentrations of 50% and 90% mortality (LC_50_ and LC_90_) were calculated using Probit analysis (29).

## Results

### Yields and chemical constituents of essential oil

The yield of essential oil was 0.5% (w/v) based on fresh weight. The essential oil was yellowish with a distinct sharp odor. Forty-six constituents in the essential oil of *P. orientalis* were identified corresponding to 97.88% of the total oil (Table 1). The major components of *P. orientalis* oil were identified as α-Pinene (20.17%), 3-Carene (14%) and Cedrol (9.51%). Other minor constituents were found to be β-Thujene (7.85%), Terpinolene (6.56%) and α-Terpinyl acetate (4.38%).

**Table 1.** Chemical constituents of leaf essential oil from *Platycladus orientalis*

**Constituents^a^**	**RI^b^**	**Composition%**
**Tricyclene**	922	0.26
**α-Pinene**	938	20.17
**α-Fenchene**	950	1.71
**β-Thujene**	966	7.85
**β-Pinene**	979	3.88
**3-Carene**	998	14.00
**2-Carene**	1006	0.77
**D-sylvestrene**	1024	0.50
**Limonene**	1032	2.74
**β-Ocimene**	1038	0.14
**α-Ocimene**	1041	0.08
**γ-Terpinene**	1056	1.73
**cis-Thujane-4-ol**	1062	0.15
**Terpinolene**	1083	6.56
**1,3,8-p-Menthatriene**	1110	0.05
**cis-p-Menth-2-en-1-ol**	1119	0.28
**(4E,6Z)-allo-Ocimene**	1130	0.02
**Borneol**	1164	0.06
**Terpinene-4-ol**	1176	2.95
**α-Terpineol**	1191	0.40
**cis-Piperitol**	1199	0.18
**Fenchyl acetate**	1225	0.06
**cis-Geraniol**	1228	0.01
**Citral**	1270	0.03
**L-bornyl acetate**	1282	1.89
**α-Terpinyl acetate**	1346	4.38
**Nerol acetate**	1366	0.42
**Geraniol acetate**	1383	0.57
**β-Elemene**	1389	0.82
**α-Cedrene**	1410	1.23
**Caryophyllene**	1424	4.32
**gamma-Elemene**	1433	0.63
**α-Caryophyllene**	1455	3.34
**Germacrene D**	1478	2.47
**β-Bisabolene**	1507	0.11
**δ-Cadinene**	1525	0.38
**Elemol**	1548	0.94
**Nerolidol**	1566	0.05
**Caryophyllene oxide**	1581	0.08
**Cedrol**	1602	9.51
**γ-Eudesmol**	1634	0.75
**Cedryl acetate**	1760	0.42
**8,15-Pimaradiene**	1890	0.02
**Hexadecanoic acid**	1971	0.02
**Pimara-7,15-dien-3-ol**	2250	0.78
**Totarol**	2301	0.20
**Total**	97.88	

a Compounds listed in order of elution from a HP-1 MS column

b Retention Indices as determined on HP-1MS using the homologous series of n-alkanes (C_8_–C_24_)

### Mosquito larvicidal activity of essential oil

The larvicidal activity of leaf oil from *P. orientalis* against *An. stephensi* and *Cx. pipiens* under laboratory conditions are shown in Table 2. Among the five concentrations tested, the dosage of 80ppm could induce more than 90% mortality in both the species.

**Table 2.** Parameters of probit regression lines of *Platycladus orientalis* oil against the larvae of *Anopheles stephensi* and *Culex pipiens*

**Species**	**A**	**B±SE**	**LC_50_, 95% C.I.**	**LC_90_, 95% C.I.**	**χ^2^ (df)**	**P-value**
***An. stephensi***	−1.77	1.66±0.159	11.51	67.81	5.9 (3)	>0.05
***Cx. pipiens***	−1.95	1.53±0.397	18.60	127.24	20.9 (3)	<0.05

A: y-intercept, B: The slope of the line, SE: Standard error,

LC_50_, 95% CI: Lethal concentration causing 50% mortality and its 95% confidence interval,

LC_90_, 95% CI: Lethal concentration causing 90% mortality and its 95% confidence interval

χ^2^= heterogeneity about the regression line, df: degree of freedom

p= represent heterogeneity in the population of tested

**Fig. 1.**
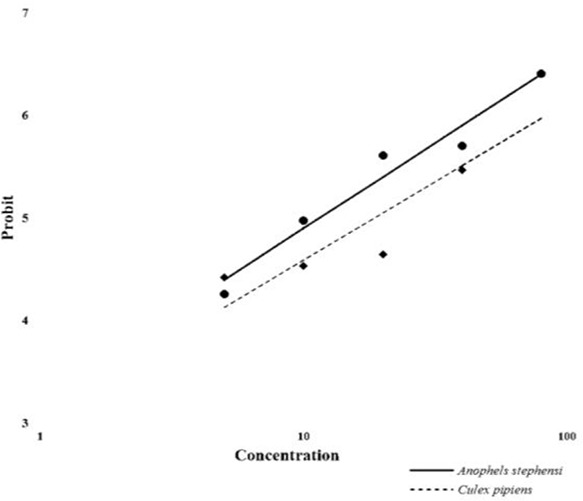
Probit regression line of *Anopheles stephensi* and *Culex pipiens* exposed to different interval concentrations of essential oil from fresh leaves of *Platycladus orientalis*

The mortality rates in the control groups were lower than 5% in all concentrations. The LC_50_ and LC_90_ values against *An. stephensi* and *Cx. pipiens* larvae were 11.51, 67.81 ppm and 18.60, 127.24ppm after 24h, respectively. Among different concentrations tested, there were no significant differences in larval mortality between the two species (P> 0.05). In regression line a positive correlation was observed between the leaf oil concentrations and mortality rates (Fig. 1).

## Discussion

The yield of oil obtained of *P. orientalis* leaves was 0.5% (w/v). This amount is similar to recent study (17) but relatively higher than other studies (22, 25, 30, 31). According to our results, α-Pinene (20.17%), 3-Carene (14%) and Cedrol (9.51%) are known as the main compounds of the oil. Similar to those previously reported (17, 26, 30, 31), the major constituents were α-pinene, 3-carene and cedrol. However, there were differences in the amount of the main components. The differences in the number of compositions in the above-mentioned studies may be due to the collection time and geographic factors (26).

Essential oils obtained from plants have been studied as a natural compound potentially used as an alternative to common synthetic insecticides. Earlier authors reported that the larvicidal activity of essential oils of various aromatic plants with LC_50_ values ranging from 24.27 to 105.4ppm and 20.61 to 311.2ppm against the larvae of *An. stephensi* and *Cx. pipiens*, respectively (12, 31–37). The leaf oil of *P. orientalis* was very effective against *An. stephensi* and *Cx. pipiens* with LC_50_ values of 11.51 and 18.60ppm after 24h, respectively, which were much lower than those of the plants studied earlier.

In the present investigation, the dosage of 80ppm was sufficient to cause 100% larval mortality against the larvae of both species after 24h. Similarly, the toxicity of *T. orientalis* and *Chamaecyparis obtusa* oils were tested against 4^th^-instar larvae of *Aedes aegypti* and *Cx. pipiens* after 24h and they reported 100% larval mortality when treated with 400ppm of both oils (23).

According to proposed categories of larvicidal activity of plant essential oils against mosquito larvae, essential oil of *P. orientalis* can be considered as active (38) to very active (39) plant.

## Conclusion

Essential oils of *P. orientalis* are promising in mosquito control. These findings could be useful in search for newer, safer, and more effective natural larvicidal compounds against disease-vector mosquitoes. Further studies must be conducted to describe the mode of action of each constituent separately also its effects on non-target organisms.
